# Evaluation of *in silico* designed inhibitors targeting MelF (Rv1936) against *Mycobacterium marinum* within macrophages

**DOI:** 10.1038/s41598-019-46295-5

**Published:** 2019-07-12

**Authors:** Renu Dharra, V. S. Radhakrishnan, Tulika Prasad, Zoozeal Thakur, Jeffrey D. Cirillo, Abhishek Sheoran, Amit K. Pandey, Mahesh Kulharia, Promod K. Mehta

**Affiliations:** 1 Centre for Biotechnology, Maharshi Dayanand University (MDU), Rohtak, 124001 India; 2 Advanced Instrumentation Research and Facility (AIRF) and Special Center for Nano Science (SCNS), Jawaharlal Nehru University (JNU), New Delhi, 110067 India; 3 Department of Microbial and Molecular Pathogenesis, Texas A&M Health Science Center, College Station, TX 77843-1114 USA; 4 Department of Statistics, Ramanujan College, University of Delhi, New Delhi, 110019 India; 5 Translational Health Science and Technology Institute (THSTI), Faridabad, 121001 India; 6 School of Basic and Applied Science, Central University of Punjab, Bathinda, 151001 India

**Keywords:** Infectious diseases, Bacteriology

## Abstract

We recently identified inhibitors targeting *Mycobacterium marinum* MelF (Rv1936) by *in silico* analysis, which exhibited bacteriostatic/bactericidal activity against *M. marinum* and *M. tuberculosis in vitro*. Herein, we evaluated the effect of best four inhibitors (# 5175552, # 6513745, # 5255829, # 9125618) obtained from the ChemBridge compound libraries, on intracellular replication and persistence of bacteria within IFN-γ activated murine RAW264.7 and human THP-1 macrophages infected with *M. marinum*. Inhibitors # 5175552 and # 6513745 significantly reduced (*p* < 0.05) the intracellular replication of bacilli during day 7 post-infection (p.i.) within RAW264.7 and THP-1 macrophages infected at multiplicity of infection (MOI) of ~1.0. These observations were substantiated by electron microscopy, which revealed the protective effect of # 5175552 in clearing the bacilli inside murine macrophages. Strikingly, # 6513745 displayed synergism with isoniazid against *M. marinum* in murine macrophages, whereas # 5175552 significantly suppressed (*p* < 0.05) the persistent bacilli during day 10–14 p.i. in infected RAW264.7 and THP-1 macrophages (MOI of ~ 0.1). Moreover, # 5175552 and # 6513745 were non-cytotoxic to host macrophages at both 1X and 5X MIC. Further validation of these inhibitors against *M. tuberculosis*-infected macrophages and animal models has potential for development as novel anti-tubercular agents.

## Introduction

Tuberculosis (TB) is a leading public health problem. In 2017, approximately 10 million people developed TB throughout the world^1^. There were ~1.3 million deaths among HIV-negative individuals and ~0.3 million deaths among HIV-positive co-infected TB individuals. India accounted for the highest TB burden (27%), including MDR (multi-drug resistant)-TB followed by China and Indonesia. The U.S Food and Drug Administration (FDA) has recently recommended the use of two drugs, *i.e*. bedaquiline and delamanid for individuals diagnosed with MDR-TB, extensively-drug resistant TB (XDR-TB) and totally-drug resistant TB. However, high doses of antibiotics, long duration of treatment, failure to kill persistent bacteria effectively by the currently used anti-tubercular drugs (ATDs) and the emergence of drug-resistant forms of *Mycobacterium tuberculosis* necessitates to immediately design new drugs, which shall be effective against all forms of TB^2^. Various strategies are employed to design ATDs, including the whole cell screening (WCS), target based screening (TBS) and the computer aided drug designing^2,3^. In WCS, cell-based high throughput screening (HTS) of compound libraries is used to identify inhibitors with whole-cell activity against *M. tuberculosis in vitro*^4^. On the contrary, in the genome-derived TBS strategy, compounds that inhibit the biochemical function of a target are identified by HTS of compound libraries or by structure-based drug designing but it does not assure the druggability of target^5,6^. Though WCS has been more successful in giving possible hits as TBS does not take into consideration the poor penetration and efflux problems, it lacks precise target knowledge^4^. Virtual ligand screening (VLS) is also considered as an effective tool for *in silico* screening of large compound libraries and small molecule chemical libraries for obtaining the potential leads against biological targets^7,8^.

The designing of new ATDs is considerably difficult primarily due to slow growth of *M. tuberculosis*. *M. marinum*, a relatively fast-grower has recently been shown to be a useful model (which requires BSL-2 facility) to evaluate the activity of ATDs within infected macrophages^9^. Zebrafish-*M*. *marinum* infection model has also been employed for evaluating ATDs *in vivo* as a HTS system^10^. Interestingly, mycobacterial *mel2* locus (Rv1936-1941) is *M. marinum* and *M. tuberculosis* specific and absent in other pathogenic and nonpathogenic mycobacterial species, which can tolerate reactive oxygen species (ROS) and reactive nitrogen species (RNS) stress response^8,11,12^. Notably, *M. marinum melF* of *mel2* locus displayed a high similarity to *luxA* of *Vibrio harveyi*, which has a significant role in resistance to ROS in bioluminescent bacteria^11^. *M. marinum melF* mutants were shown to be defective for bacterial growth in activated murine macrophages and revealed a reduced bacterial growth at late stages in the C57BL/6 mouse footpad model of infection^12^. Furthermore, the association of *mel2* locus in resistance to ROS has been documented during the persistence stage of *M. tuberculosis* infection in mice^13^. Using *in silico* VLS and TBS approach, we identified potent inhibitors targeting *M. marinum* MelF (~84 kDa, a dimeric protein) that significantly suppressed the flavin oxidoreductase activity of MelF and exhibited bacteriostatic/bactericidal effect against *M*. *marinum* and *M*. *tuberculosis* in an *in vitro* system^7^, however, the effect of these inhibitors against mycobacterium-infected macrophages was not examined. Indeed, macrophages are the key effectors cells to control mycobacterial infections and that also have a niche for their replication^14,15^, which produce antibacterial ROS and RNS molecules through NADPH oxidase and inducible nitric oxide synthase^8,16^. Mostly, mycobacteria are resistant to ROS, but RNS display bacteriostatic/bactericidal effect within activated macrophages. We hypothesized that targeting and inhibiting mycobacterial MelF (with anti-ROS/RNS activity) would facilitate efficient clearance of bacteria from the invading macrophages. Therefore, this study was designed to evaluate the effect of *in silico* designed inhibitors targeting MelF on entry, intracellular replication and persistence of bacteria within IFN-γ activated macrophages infected with *M. marinum*.

## Results

In preliminary studies, the production of ROS levels in all the experiments were evaluated in IFN-γ activated RAW264.7 and phorbol 12-myristate 13-acetate (PMA)-treated THP-1 macrophages by measuring H_2_O_2_ production^12^, which was found to be in the range of 10–12 µM (data not shown).

### Evaluation of cytotoxicity of the inhibitors in RAW264.7 and THP-1 macrophages

**Figure 1:**
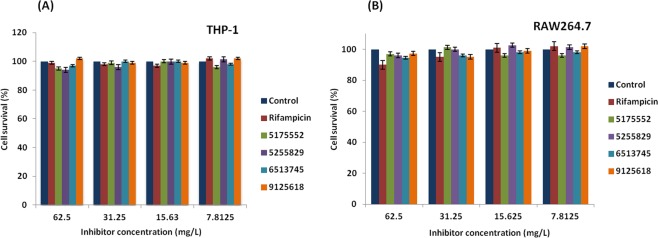
Determination of cytotoxicity of inhibitors by MTT assay: (**A**) Survival of human THP-1 macrophages and (**B**) murine RAW264.7 macrophages in presence of varying concentrations of inhibitors. Rifampicin was taken as a positive control. Data represent mean ± SD from two independent experiments done in triplicates.

The *in silico* designed inhibitors of Chembridge compound libraries are detailed in a recent report^7^. Among these, the best four inhibitors (# 5175552, # 5255829, # 6513745 and # 9125618) were evaluated for cytotoxicity in RAW264.7 and THP-1 macrophages in this study. We chose 1X and 5X MIC (minimum inhibitory concentration) values of inhibitors ranging from 1.56–62.5 µg/mL to examine cytotoxicity by MTT (3-(4,5-dimethyl thiazol-2yl)-2, 5-diphenyl tetrazolium bromide) assay (Fig. 1). It was observed that >90% of both THP-1 and RAW264.7 macrophages survived at a concentration of 62.5 µg/mL, while >95% of these cells survived at lesser concentrations (7.8–31.25 µg/mL, Fig. 1). Interestingly, each inhibitor was found to be non-cytotoxic (as viability of cells was >95%) even up to 5 X MIC.

### Cell association/entry of *M. marinum* within activated murine RAW264.7 macrophages

To mimic the actual immune response elicited during an infection, macrophages were activated with IFN-γ before infection to create stringent growth conditions for *M. marinum* inside the host macrophages^12^. We evaluated the entry of WT (wild-type) *M. marinum*, *M. marinum* ΔMelF (MelF mutant) and WT + inhibitors at 1X MIC in activated murine RAW264.7 macrophages. It was observed that 9.75% of the WT inoculum could associate/enter within RAW264.7 cells at an MOI of ~ 0.2 (Table 1). On the other hand, significantly lesser (*p* < 0.05) inoculum of ΔMelF, WT + # 5175552 and WT + # 6513745 could associate/enter within murine macrophages as compared to WT alone. It is pertinent to mention that we did not incorporate the aminoglycoside antibiotic such as amikacin/gentamicin in such assays to kill extracellular bacteria for determining the true entry/invasion^17–19^, since presence of amikacin/gentamicin would otherwise give incorrect results. Therefore, we used the term cell association (entry) for such assays.

**Table 1:** Effect of inhibitors on % cell association (entry) of *M. marinum* inside the activated RAW264.7 cells within 30 minutes.

Group	CFU (mean ± SD)	% Cell Association*
WT *M. marinum*	1.91 × 10^4^ ± 0.09 × 10^4^	9.75 ± 0.43
ΔMelF	1.03 × 10^4^ ± 0.07 × 10^4^	5.24 ± 0.36
WT + RIF	1.67 × 10^4^ ± 0.03 × 10^4^	8.50 ± 0.15
WT + # 5175552	1.11 × 10^4^ ± 0.09 × 10^4^	5.65 ± 0.46
WT + # 5255829	1.51 × 10^4^ ± 0.19 × 10^4^	7.66 ± 0.99
WT + # 6513745	0.99 × 10^4^ ± 0.09 × 10^4^	5.05 ± 0.46
WT + # 9125618	1.95 × 10^4^ ± 0.05 × 10^4^	9.92 ± 0.25

*Inoculum pertaining to MOI of ~ 0.2 (1.96 ± 0.265 × 10^5^) was used. Data represent mean ± SD of two independent experiments done in triplicates.

Similarly, we did not incorporate amikacin/gentamicin in cell culture medium to study the effect of inhibitors on intracellular replication/persistence of *M. marinum* bacilli within macrophages.

### Intracellular replication of *M. marinum* within IFN-γ activated macrophages

**Figure 2:**
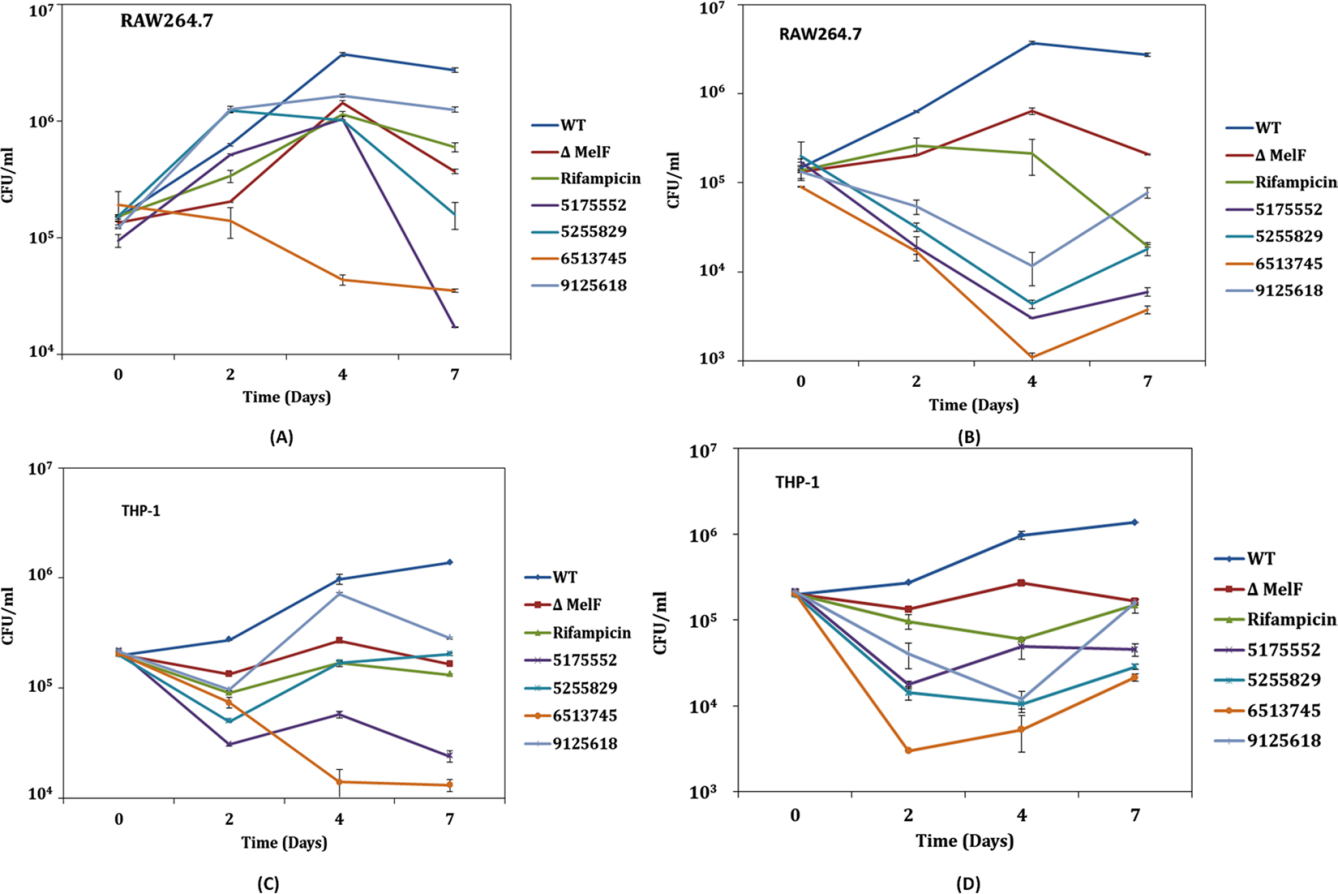
Intracellular replication of *M. marinum*-infected macrophages at MOI of ~1.0 during 7 days experiment: Intracellular replication of WT *M. marinum*, ΔMelF, Rifampicin treated WT (Rifampicin) and inhibitor-treated WT (# 5175552, # 5255829, # 6513745, # 9125618) within IFN-γ activated RAW264.7 and THP-1 macrophages at 1X MIC (**A**,**C**) and 5X MIC (**B**,**D**) on day 0, 2, 4 and 7 p.i. A significant difference (*p* < 0.05) in intracellular replication was observed between the WT and inhibitor-treated WT (# 5175552, # 5255829 and # 6513745) on day 4 and 7 p.i. Data represent mean ± SD of two independent experiments done in triplicates.

The inhibitors permeated the mycobacterial cells within activated RAW264.7 and THP-1 macrophages at both 1X and 5X MIC (Fig. 2 and Suppl. Table 1). By day 4 p.i., we observed 25.2-fold increase in CFU for WT within RAW264.7 cells, which declined thereafter (Fig. 2A,B). We compared the CFU data of ΔMelF and of WT + inhibitors, with the WT alone. There was 2.5- and 7.4-fold decrease in CFU for ΔMelF on day 4 and 7 p.i., respectively in RAW264.7 macrophages (Fig. 2A,B). However, 3.5-fold decrease in CFU was observed for inhibitor # 5175552 (1X MIC) and 1.9 log decrease in CFU for # 6513745 (1X MIC) within RAW264.7 macrophages on day 4 p.i., compared to WT alone. In a similar manner, 2.2 and 1.9 log differences in CFU were observed for # 5175552 and # 6513745, respectively at 1X MIC on day 7 p.i. However, at 5X MIC, # 5175552 and # 6513745 displayed 3 and 3.5 log differences in CFU, respectively, as compared to WT alone on day 4 p.i.; while 2.6 and 2.8 log decrease in CFU were observed for # 5175552 and # 6513745, respectively within RAW264.7 macrophages on day 7 p.i. thus indicating dose-dependent inhibition of *M. marinum* bacilli by these inhibitors.

We observed 3.6- and 8.3-fold decrease in CFU for ΔMelF on day 4 and 7 p.i., respectively, compared to WT in THP-1 macrophages (Fig. 2C,D). However, at 1X MIC, CFU reduced to 1.8 and 2 log for inhibitors # 5175552 and # 6513745, respectively, as compared to WT alone on day 7 p.i., while at 5X MIC, 1.5 and 1.8 log decrease in CFU, respectively were observed within THP-1 macrophages on day 7 p.i.

### Synergism of # 6513745 with isoniazid (INH) against *M. marinum* within activated RAW264.7 macrophages

**Figure 3:**
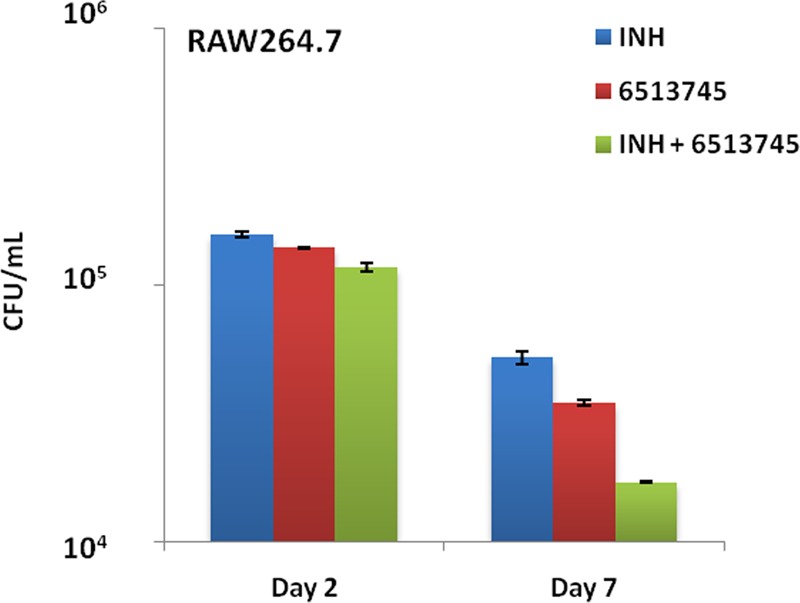
Synergism of # 6513745 + INH on intracellular replication of *M. marinum* within activated RAW264.7 macrophages: Growth inhibition of WT *M. marinum* within murine macrophages at an MOI of ~1.0 in presence of INH (6.25 µg/mL) alone, # 6513745 alone (6.25 µg/mL) and # 6513745 (6.25 µg/mL) + INH (6.25 µg/mL) on day 2 and 7 p.i. Data represent mean ± SD of two independent experiments done in triplicates.

Notably, the inhibitor # 6513745 displayed a synergistic effect with INH against *M. marinum* within activated RAW264.7 macrophages on day 7 p.i. as INH + # 6513745 revealed a significantly diminished (*p* < 0.05) bacillary load than INH alone (Fig. 3). Moreover, no cytotoxicity was observed on host macrophages (data not shown).

### Intracellular survival/killing of persistent *M. marinum* by the inhibitors within activated macrophages

**Figure 4:**
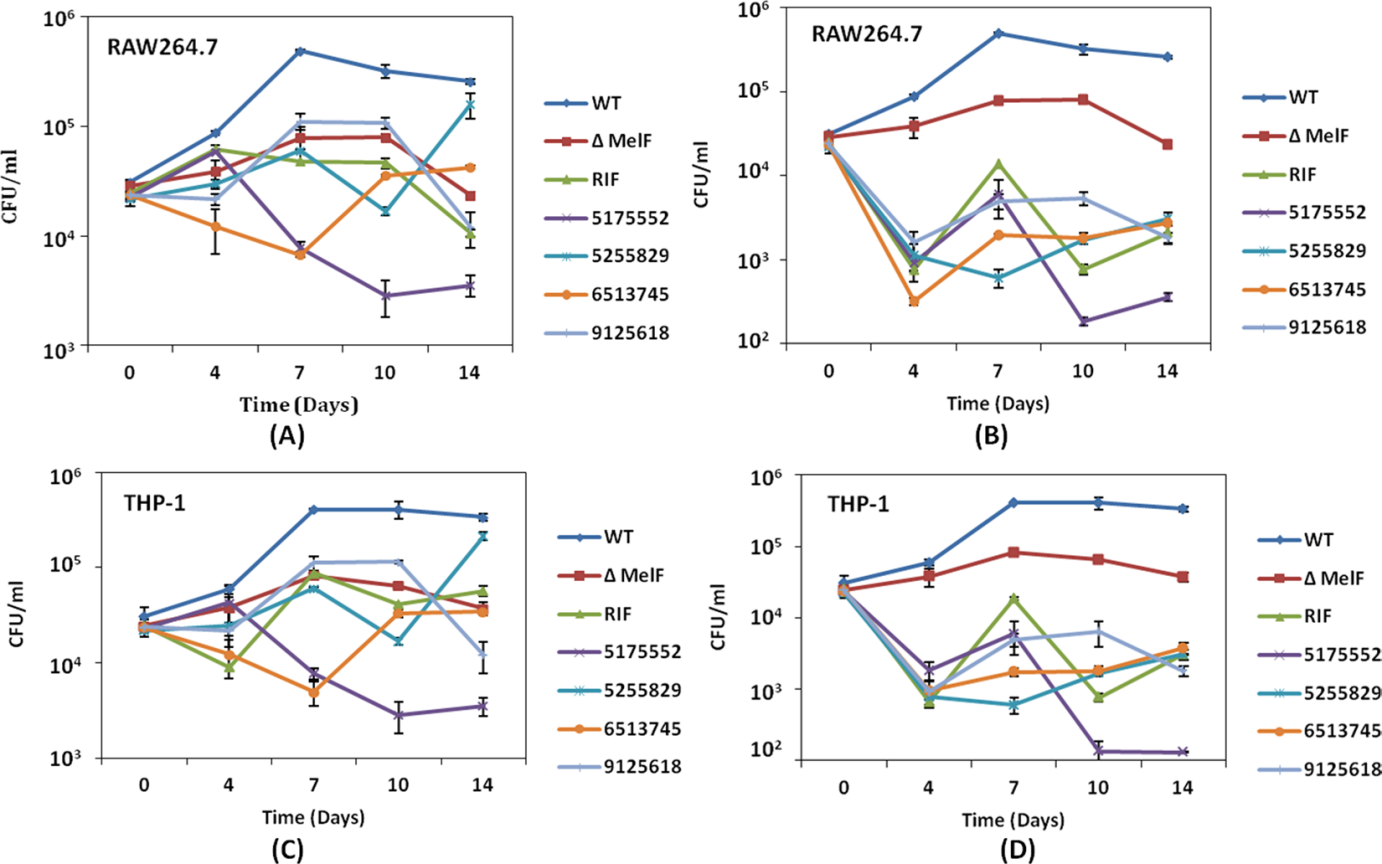
Persistence of *M. marinum*-infected macrophages with MOI of ~ 0.1 during 14 days experiment: Intracellular survival/persistence of WT *M. marinum*, ΔMelF, Rifampicin-treated WT (Rifampicin) and inhibitor-treated WT (# 5175552, # 5255829, # 6513745 and # 9125618) within IFN-γ activated RAW264.7 and THP-1 macrophages at 1X MIC (**A**,**C**) and 5X MIC (**B**,**D**) on day 7, 10 and 14 p.i. A significant difference (*p* < 0.05) in intracellular survival/persistence was observed between the WT and inhibitor-treated WT (# 5175552, # 6513745 and # 9125618) on day 7, 10 and 14 p.i. Data represent mean ± SD of two independent experiments done in triplicates.

During the 14 days persistence experiment, WT bacilli increased 15.7- and 13.2-fold by day 7 p.i. within activated RAW264.7 and THP-1 macrophages, respectively and later the bacillary load declined and became steady during day 10–14 p.i. (Fig. 4A–D). On the other hand, ΔMelF revealed 6.3-, 4- and 10.9-fold decrease in CFU within RAW264.7 macrophages on day 7, 10 and 14 p.i., respectively as compared to WT alone (Fig. 4A,B and Suppl. Table 2). Similarly, 4.9-, 6.3- and 9.1-fold decrease in CFU was observed for ΔMelF within THP-1 macrophages on day 7, 10 and 14 p.i., respectively (Fig. 4C,D). Interestingly, a dose-dependent inhibitory effect of # 5175552 was observed in both activated RAW264.7 and THP-1 macrophages since greater (2.85 and 3.4 log) reductions in persistent bacilli were observed with # 5175552 at 5X MIC on day 14 p.i. as compared to 1X MIC (Fig. 4B,D).

Strikingly, the inhibitory effect of # 6513745 was less pronounced as compared to # 5175552 in persistence experiments within both RAW264.7 and THP-1 macrophages on day 10 and 14 p.i. (Fig. 4A–D), thus implying that this inhibitor was less effective against the persistent bacilli as compared to its effect on intracellular replication. Overall, #5175552 seems to be the best inhibitor (followed by # 6513745), since it efficiently inhibited intracellular replication and cleared persistent *M. marinum* bacilli at both 1X and 5X MIC.

### Electron microscopy

**Figure 5:**
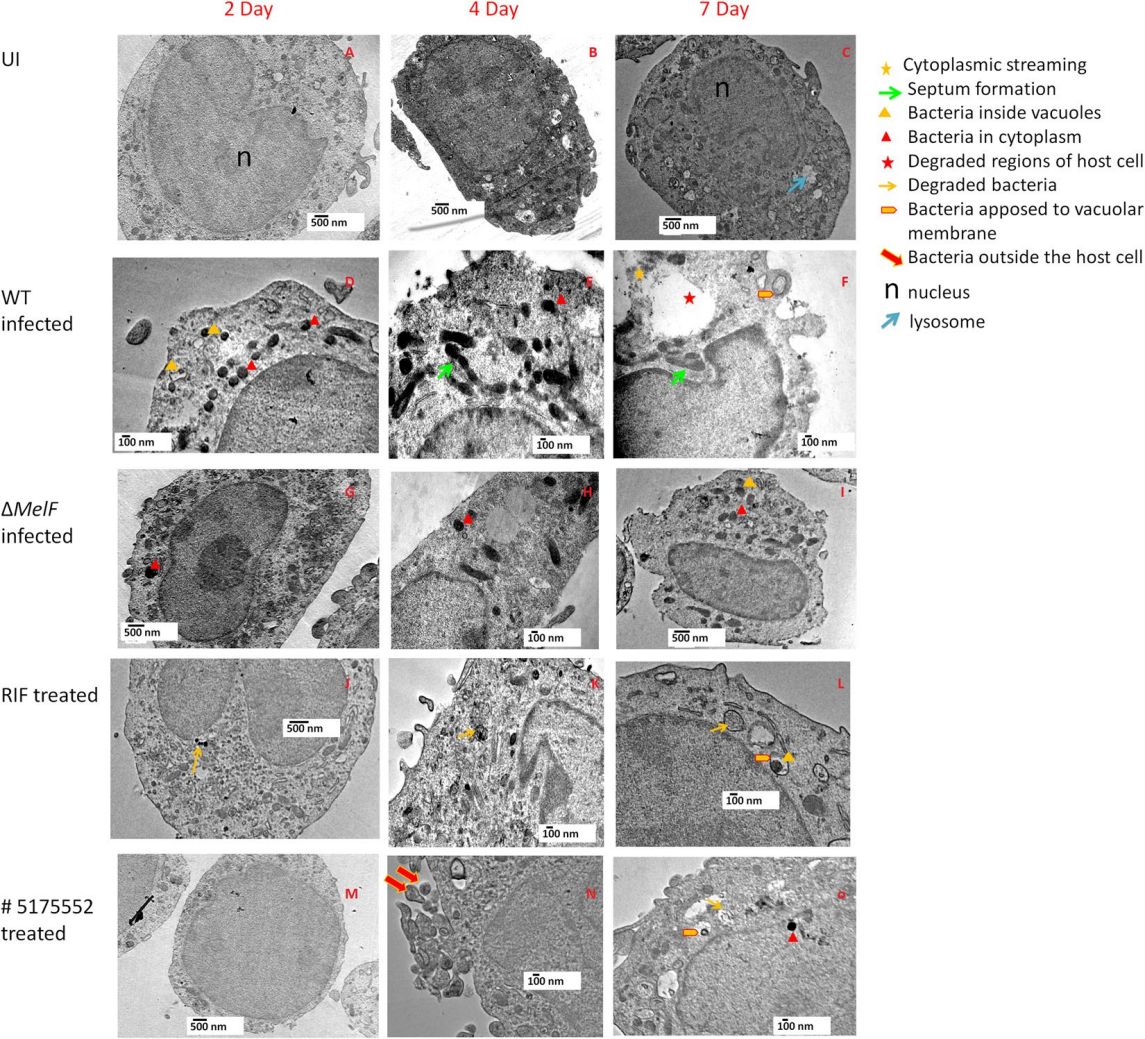
TEM analysis: TEM images were acquired on day 2, 4 and 7 p.i. after infection of murine IFN-γ treated RAW264.7 macrophages with WT *M. marinum* at an MOI of ~1.0. Majority of bacilli were found in vacuoles and cytoplasm, distinct from lysosomes in WT-, ΔMelF-, Rifampicin (RIF)-treated WT- and # 5175552-treated WT-infected macrophages (**D**–**O**). Bacilli were also found to be tightly apposed to the vacuolar membranes on day 7 p.i. in WT-, RIF-treated WT- and #5175552-treated WT-infected macrophages (**F**,**L**,**O**). **(A–C**) Uninfected RAW264.7 macrophages (UI) on day 2, 4 and 7 p.i. Macrophages were healthy with intact cell membrane throughout day 2–7 (**A**–**C**). UI macrophages showed distinct plasma membrane, vacuole, nucleus and lysosome. **(D–F**) WT *M. marinum* infected RAW264.7 cells. WT infected macrophages showed damaged plasma membrane on day 4 and 7 p.i. (**E**,**F**). On day 7 p.i., ruptured plasma membrane, nuclear condensation and cytoplasmic streaming were observed with extrusion of intracellular bacteria and degraded cellular materials (**F**). Notably, WT *M. marinum* bacillary replication was evident from the appearance of bacterial septa on day 4 and 7 p.i. (**E**,**F**). **(G**–**I**) *M. marinum* ΔMelF infected RAW264.7 cells. ΔMelF infected macrophages showed protection to host cells as evident from lesser cellular damage. However, on day 7 p.i., the plasma membrane of host cell showed a punctate appearance. As compared to WT, lesser number of intracellular bacilli were observed on day 2, 4 and 7 p.i. (**G**,**H**,**I**). Also, the host cell membrane integrity remained intact with no visible damage even on day 4 and 7 p.i. (**H**,**I**). **(J–L**) RIF-treated WT infected RAW264.7 cells. Lesser number of *M. marinum* bacilli was observed in RIF-treated WT infected host cells than with WT alone on day 2, 4 and 7 p.i. (J-L). Bacterial debris were found to be present both within and outside the vacuoles on day 2, 4 and 7 p.i. In addition, cell membrane of host cells remained intact and the macrophages appeared relatively healthier, as compared to that with WT alone (**J**–**L**). (**M**–**O**) # 5175552-treated WT infected RAW264.7 cells. Lesser intracellular bacilli were observed in # 5175552-treated WT infected host cells and the macrophages appeared relatively healthier as compared to that with WT, ΔMelF and RIF-treated WT on day 2, 4 and 7 p.i. Interestingly, more extracellular bacilli than intracellular bacilli were distinctly visible on day 4 p.i. (**N**). Furthermore, bacterial debris were observed in both vacuoles and cytoplasm of infected macrophage on day 7 p.i. (**O**). Respective scale bar has been indicated in each micrograph.

Transmission electron microscopy (TEM) clearly revealed the presence of *M. marinum* bacilli inside both vacuoles and cytoplasm of macrophages infected with WT, ΔMelF, RIF-treated WT and # 5175552-treated WT (Fig. 5). Strikingly, distinct formation of bacterial septa was observed on day 4 and 7 p.i. (shown by arrowheads in Fig. 5E,F) in WT *M. marinum*-infected RAW264.7 macrophages, which indicated bacterial replication inside the host cell. On day 7 p.i., degraded regions of the host cell infected with WT were clearly visible along with simultaneous lysis of the host cell membrane, which led to cytoplasmic streaming and extrusion of bacilli into the extracellular space (Fig. 5F). In contrast, features such as large number of intracellular bacteria, septum formation, host cell lysis and cytoplasmic streaming were not observed in the uninfected macrophages (UI, Fig. 5A–C) and the macrophages infected with ΔMelF (Fig. 5G–I), RIF-treated WT (Fig. 5J–L) and # 5175552-treated WT (Fig. 5M–O). Also, *M. marinum* bacilli were found to be tightly apposed to the vacuolar membranes in WT, RIF- and #5175552-treated WT on day 7 p.i. (Fig. 5F,L,O).

In the ΔMelF-infected macrophages, lesser number of bacilli were observed on day 2, 4 and 7 p.i. (Fig. 5G–I), as compared to that with WT, which corroborates with the CFU data. The membrane integrity remained intact, without any visible damage to the host cell till day 7 p.i. (Fig. 5G–I). Similarly on day 2, 4 and 7 p.i., RIF- and # 5175552-treated WT infected macrophages showed intact nucleus and host cell membrane integrity with presence of bacillary debris and lesser number of intracellular bacilli (Fig. 5J–O), as compared to that with WT. Of note is the fact that within # 5175552-treated WT infected macrophages, more number of bacilli was observed outside the host cell rather than inside the cell on day 4 p.i., (Fig. 5N). In addition, bacterial debris was observed in both vacuoles and cytoplasm on day 7 p.i. (Fig. 5O). These observations clearly indicate the protective effect of RIF- and #5175552-treated WT in clearing the *M. marinum* bacilli within RAW264.7 macrophages. To add, # 5175552 exhibited better clearance of *M. marinum* bacilli than RIF-treated WT.

## Discussion

ROS and RNS are the most effective antimycobacterial molecules generated by the host macrophages during infection. Infection of Phox−/− and NOS−/− mice as well as bone marrow-derived macrophages infected with *M. tuberculosis* clearly suggest that the *mel2* locus imparts pathogenesis through its ability to resist host ROS stress^8,13^. *M. tuberculosis* is also constantly exposed to endogenous ROS including the production of superoxide radicals during normal aerobic respiration^20^. We previously demonstrated that the *M. marinum* MelF mutant was defective for growth in activated J774A.1 cells, which was annulled by inhibitors of ROS scavengers or nitric oxide synthase inhibitors^12^. In this study, we examined the efficacy of *in silico* designed inhibitors targeting mycobacterial MelF on intracellular replication and persistence of bacteria within IFN-γ activated RAW264.7 and THP-1 macrophages-infected with *M. marinum*^7^.

Comparable to *M. marinum* strain M-infected RAW264.7 and THP-1 macrophage model of this study, intracellular replication of *M. marinum* BAA-535 strain within J774A.1 macrophages was previously used to assess the inhibitory activity of compounds targeting isocitrate lyase (ICL, Rv0467)^9^. IMBI-3, a competitive inhibitor was found to be the most effective compound with an inhibition constant (Ki) of 1.85 μM^9^. Interestingly, # 5175552 used in this study is also a competitive inhibitor targeting flavin oxidoreductase of MelF with Ki of 68 μM, while # 6513745 is a non-competitive inhibitor with Ki of 18 μM^7^. Furthermore, Liu *et al*.^9^ demonstrated the susceptibility of *M. marinum* BAA-535 strain to14 of first-line and second-line ATDs (both intracellular and extracellular), thus establishing the possibility of using *M. marinum* BAA-535 to evaluate anti-TB activity within murine macrophages. Similar to non-replicating *M. tuberculosis* bacilli which are resistant to INH, *M. marinum* BAA-535 bacilli persisting inside the J774A.1 macrophages were also found to be resistant to INH, but not to RIF; thus proposing it as a surrogate model to assess the activity of inhibitors on persistent *M. tuberculosis*^9^. In this study, we also evaluated the efficacy of MelF inhibitors against the persistent *M. marinum* strain M within RAW264.7 and THP-1 activated macrophages infected at low MOI of ~ 0.1 during the duration of 14 days p.i.

Similarly, a BSL-2 compatible model for testing the efficacy of drugs has been developed using THP-1 cells-passaged *gfp* expressing *M. tuberculosis* mc^2^6206 (Δ*panCD*, Δ*leuCD*), which formed densely packed *M. tuberculosis*/macrophage aggregate structures. *M. tuberculosis* mc^2^6206, a derivative of H_37_Rv strain contains all but four genes, *i.e. panCD* and *leuCD*, which are not involved in virulence and drug resistance^21,22^. The MIC of standard ATDs against this auxotrophic strain was determined and its requirement of l-leucine and pantothenate for replication did not affect the drug susceptibility as compared to the parent H_37_Rv strain. However, the macrophage-passaged *M. tuberculosis* might revert back to the virulent BSL-3 compatible mycobacterium, which would then involve a high risk for the handling personnel. Moreover, this auxotroph does not replicate during macrophage infection thus a higher MOI is needed to mimic the intracellular replication observed with the WT *M. tuberculosis*^21^. Although *M. smegmatis* (a fast grower that lacks most *M. tuberculosis* virulence genes) and *M. bovis* BCG also surrogate *M. tuberculosis* and can be handled in BSL-2 conditions; these mycobacterial species have limitations. For example, *M. bovis* does not naturally infect humans and *M. smegmatis* has different antibiotic resistance profiles and is killed by the activated macrophages^21,23^. Therefore, *M. marinum*-infected macrophages used in this study seem to be an alternative useful model to assess the activity of inhibitors.

Our data on entry and intracellular replication of WT *M. marinum* and ∆MelF within activated RAW26.7 macrophages (Table 1 and Fig. 2) are in agreement with the previous reports^12,19,24^, however, intracellular replication was previously examined up to 48 h p.i. only. Bacterial multiplication of WT *M. marinum* within vacuoles and cytoplasm of RAW264.7 cells, septum formation as well as host cell lysis and cytoplasmic streaming on day 4 and 7 p.i. observed by TEM analysis (Fig. 5) are in accordance with the earlier observations made with *M. tuberculosis*-infected J774A.1 macrophages and the cultured human lung microvascular endothelial cells^17,18^. Interestingly, *M. marinum* bacilli were found to be tightly apposed to the vacuolar membranes of RAW264.7 macrophages infected with WT, RIF- and # 5175552-treated WT on day 7 p.i., which also corroborates with the earlier report^25^, wherein the virulent mycobacteria survived and probably replicated within a unique tight vacuole in the infected macrophages of the lungs. We demonstrated that the inhibitors # 5175552 and # 6513745 were quite effective in inhibiting the intracellular replication during 7-day and clearing the bacillary persistence during 14-day experiments in IFN-γ activated RAW264.7 macrophages (Figs 2, 4), which was dose-dependent as higher inhibition/clearance of bacillary load was observed at 5X MIC than at 1X MIC. Although these inhibitors were effective in activated THP-1 macrophages but the dose-dependent effect was not observed during 7-day intracellular bacillary replication, whereas the clearance of persisting bacilli during 14-day experiment was dose-dependent. In a similar manner, tamoxifen (a synthetic anti-estrogen) significantly reduced both drug-sensitive and drug-resistant intracellular *M. tuberculosis* bacilli within RAW264.7 macrophages in a dose-dependent manner^26^. In addition, thiosulfinate-derivative rich extract of *Allium sativum* showed better inhibitory activity than its isolated constituents against *M. tuberculosis* probably due to synergism amongst the constituents of garlic extract, which also revealed a dose-dependent effect within RAW264.7 macrophages^27^ with no cytotoxicity in host macrophages as determined by MTT assay, whereas our study demonstrated no cytotoxicity with inhibitors on RAW264.7 and THP-1 macrophages even at 5X MIC (Fig. 1). Consistent with the CFU data, the efficient clearance of intracellular *M. marinum* bacilli within RAW264.7 macrophages was also observed with inhibitor # 5175552 on day 7 p.i. as revealed by TEM analysis (Fig. 5).

Interestingly, the combination of # 6513745 with INH was significantly more effective (*p* < 0.05) in clearing the bacterial load within activated RAW264.7 macrophages on day 7 p.i. as compared to INH alone (Fig. 3), which could be due to the facilitated penetration of # 6513745 inside the *M. marinum* bacilli in presence of INH, that acts primarily on mycobacterial cell wall^28^. These findings are in agreement with our observations with # 6513745 + INH against *M. marinum in vitro*^7^. Such synergy could also be due to activation of INH through ROS generator, *i.e*. # 6513745 (by inhibiting MelF possessing anti-ROS/RNS machinery), thus leading to high susceptibility of mycobacteria within RAW264.7 macrophages. Moreover, compounds such as clofazimine and plumbagin capable of generating ROS through production of superoxide radicals, were previously shown to reduce the MIC of INH for *M. tuberculosis*^8,29^. In a similar manner, inhibitor IMBI-3 targeting ICL exhibited a synergistic effect in combination with INH against persistent *M. marinum* within J774A.1 macrophages^9^. Also, the efflux pump inhibitors such as verapamil, timcodar, thioridazine, etc. have been shown to synergize with the existing ATDs^30,31^. For example, Pieroni *et al*.^31^ synthesized a number of putative efflux inhibitors based on the structure of thioridazine. Some of those synthesized compounds revealed higher efflux inhibition, lesser cytotoxicity and synergistic bactericidal effect with the existing ATDs against *M. tuberculosis in vitro* as well as *ex vivo* (inside the infected human monocyte-derived macrophages)^31^. Furthermore, timcodar (earlier known as VX-853) displayed synergy with RIF, moxifloxacin and bedaquiline against *M. tuberculosis* infection within human THP-1 macrophages^30^.

During the long-term persistence experiments at low MOI of ~0.1 in cultured RAW264.7 and THP-1 macrophages, # 5175552 was found to be more efficacious than # 6513745 in clearing the non-multiplying persistent bacteria during day 10–14 p.i. (Fig. 4). The different effects of inhibitor # 6513745 could be due to different growth conditions, *i.e*. non-replicating versus replicating within macrophages. In a similar manner, the inhibitory activity of FDA approved bedaquiline (TMC207, diarylyquinoline) against *M. tuberculosis* in bacteriological liquid medium (7H9 broth) initially revealed a bacteriostatic phase for 7 days and later a dose-dependent bactericidal activity was observed^32^. However, the same drug initially showed a negligible bacteriostatic phase in murine peritoneal macrophages and J774 macrophages, while the bactericidal effect was noticed on day 5–7 p.i., which was probably due to low bacterial ATP levels in presence of TMC207, thus demonstrating different effects of drug in different growth conditions (*in vitro* and *ex vivo*). Moreover, pyrazinamide acts primarily on the non-multiplying persistent bacteria but is inactive against the actively replicating *M. tuberculosis* bacteria^33^.

TB treatment becomes considerably difficult due to tolerance displayed by the persistent *M. tuberculosis* bacilli towards the conventional ATDs and there is an urgent need to devise new drugs that would specifically kill the persisting bacteria located deep inside the tissues. During the persistent stage also, the bacilli are continually exposed to ROS and RNS generated as part of the host response and redox-defense mechanisms are considered vital for the survival of the mycobacterium^34^. Similar to *mel2* locus that is involved in persistence of the tubercle bacilli^13^, the sulfur metabolism and *de novo* cysteine biosynthesis pathways are involved in maintaining the redox homeostasis for the persistent tubercle bacilli^34^, which are thus suggested as potential drug targets to treat latent TB. Protein kinase G, a thioredoxin-fold containing eukaryotic like serine/threonine protein kinase of *M. tuberculosis* (MtPknG, Rv0410c) also mediates persistence under stressful conditions like hypoxia and assists in drug tolerance^35^. In an attempt to design inhibitors against MtPknG, Singh *et al*.^36^ selected six ligands by employing a sequential pharmacophore-based VLS protocol, wherein three of them exhibited significant MtPknG inhibition, while we employed the structure-based VLS approach for shortlisting MelF inhibitors^7^. Furthermore, a significant inhibition of the *M. bovis* BCG bacilli was observed in THP-1 macrophages by the compound NRB04248 with no cytotoxicity to host macrophages^36^, as observed with inhibitors # 5175552 and # 6513745 on *M. marinum*-infected THP-1 macrophages in this study. However, the effect of NRB04248 on *M. bovis* BCG-infected macrophages was examined till 48 h p.i. only^36^, whereas the long-term intracellular replication and persistence experiments were not performed. A recent trend in ATD development has also been to target the host molecules that are considered crucial for intracellular survival of *M. tuberculosis*, *e.g*. tyrosine kinase Src inhibitors significantly reduced the survival of H_37_Rv as well as MDR and XDR strains of *M. tuberculosis* within human THP-1 macrophages^37^, which may develop into host-directed ATDs.

In brief, this study demonstrates that *in silico* designed inhibitor # 5175552 (N~1~,N~1~-diethyl-N~4~-{6-methoxy-2-[2-(4-nitrophenyl)vinyl]-4-quinolinyl}-1,4-pentanediamine) targeting MelF significantly suppressed the intracellular replication and persistence of *M. marinum* within activated murine RAW264.7 and human THP-1 macrophages with no cytotoxicity to host macrophages. Similarly, # 6513745 ([3-(4-isobutoxyphenyl)-1-phenyl-1H-pyrazol-4-yl] methanol) suppressed the intracellular replication of *M. marinum* within activated macrophages and displayed synergism with INH. Validation of these findings in *M. tuberculosis*-infected macrophages and animal models in BSL-3 facility certainly needs further investigations to find the suitability of these candidates for development as anti-tubercular agents. Nonetheless, this study would undoubtedly set up new directions in designing more ROS generators, which may also improve the effectiveness of existing ATDs.

## Methods

### Bacterial strains and growth conditions

Wild-type (WT) *M. marinum* strain M and *M. marinum* melF mutant (ΔMelF) were received from Dr. Cirillo’s laboratory, Texas A & M University, College Station, TX, USA, which were grown at 33 °C for ~7 days in 7H9 Middlebrook medium supplemented with 0.5% glycerol, 10% albumin dextrose complex (ADC) and 0.05% Tween-80 (M-ADC-TW) and M-ADC-TW supplemented with 10 µg/mL of kanamycin, respectively as detailed in previous papers^7,11^. *M. marinum* colony-forming units (CFU) were enumerated on 7H10 (M-ADC) agar^7,11^, while CFU enumeration of ΔMelF was carried out on 7H10 (M-ADC) agar + kanamycin (10 µg/mL). This work was carried out in Dr. Pandey’s laboratory, after taking approval from the Departmental Committee of THSTI, Faridabad to work on *M. marinum* in biosafety-hood.

### *In silico* designed inhibitors

We employed a bi-pronged protocol for identifying the potential *M. marinum* MelF inhibitors based on the consensus between the docking results of DOCK 3.6 and Vina 1.0.2 docking methods as detailed in a recent paper^7^. Briefly, ~1 million compounds from the ChemBridge compound libraries were filtered using FAFDrugs2 to eliminate compounds with poor ADMETox indices by applying hard-filters. The sdf files of these shortlisted compounds (~ 250,000) were subjected to PDB format using Open Babel 2.2.3^38^. The compounds were subjected to VLS with Vina 1.0.2 and DOCK 3.6. Top 1000 consensus-ranked molecules were shortlisted as putative MelF inhibitors. Among these, 178 molecules were shortlisted for experimental testing based on their docking score, scaffold diversity and the manual assessment of their fit in the targeted site. These 178 inhibitors were procured from Chembridge Corp., San Diego, USA and tested for diminished flavin oxidoreductase activity against the purified *M. marinum* MelF, which were further shortlisted on the basis of MIC and MBC (minimum bactericidal concentration) values determined against *M. marinum* and *M. tuberculosis in vitro*^7^. Best four inhibitors, *i.e*. # 5175552, # 5255829, # 6513745 and # 9125618 were evaluated against the *M. marinum*-infected macrophages in this study. These inhibitors were received as dry powder in vials (each containing 5 mg). A stock solution of 5 mg/mL for each inhibitor was prepared by dissolving in 100% DMSO. The respective stock solutions were further diluted in cell culture medium to attain the desired concentrations for macrophage experiments. The final concentrations of DMSO for these inhibitors were in the range of 0.01–0.5%, which is non-cytotoxic for the cell culture.

### Maintenance of RAW264.7 and THP-1 cell lines

Murine RAW264.7 macrophages were maintained in Dulbecco’s modified eagle medium (DMEM) supplemented with 10% foetal bovine serum (FBS) and incubated at 37 °C in 5% CO_2_ with humidified atmosphere. Similarly, human THP-1 monocytic cells were maintained in RPMI-1640 medium supplemented with 10% FBS and incubated at 37 °C in 5% CO_2_ with humidified atmomsphere.THP-1 monocytic cells were treated with 10 ng/mL PMA to convert them into THP-1 macrophages. During cell association/entry assays, the culture medium was supplemented with 10% FBS. However, the culture medium supplemented with 1% FBS was used for the intracellular replication and persistence experiments in order to slow the growth of the cell culture thus permitting experiments to proceed for 7 and 14 days, respectively^17,19^. Viability of RAW264.7 and THP-1 macrophages was >95% and >80% during 7-day and 14-day experiments, respectively as assessed by trypan blue exclusion. The MIC of RIF/INH and the inhibitors were employed as reported in a previous paper (0.39 µg/mL for RIF, 6.25 µg/mL for INH, 1.56 µg/mL for # 5175552, 12.5 µg/mL for # 5255829, 6.25 µg/mL for # 6513745, 6.25 µg/mL for # 9125618)^7^. During the infection studies, cultured macrophages were maintained at 33 °C to provide optimal growth conditions for WT *M. marinum* and ΔMelF^12,39^.

### Cytotoxicity assay

Cytotoxicity of the inhibitors was studied in murine RAW264.7 and human THP-1 macrophages by MTT (3-(4,5-dimethyl thiazol-2yl)-2,5-diphenyl tetrazolium bromide) assay as described in previous papers with little modifications^7,40^. Briefly, RAW264.7 and THP-1 cells were plated at a concentration of 1 × 10^5^/well and 2 × 10^5^/well, respectively in 100 µL of cell culture medium +10% heat-inactivated FBS in 96-well plates. After overnight incubation at 37 °C in 5% CO_2_ incubator with humidified atmosphere, medium was removed and different concentrations of inhibitors were added in 100 µL of culture medium containing 10% heat-inactivated FBS. Also, various concentrations of rifampicin were included in such assays, which served as a positive control. After incubation for 20 h, cells were washed with warm cell culture medium (without FBS) and 100 μL of MTT (0.5 mg/mL) was added. The plates were further incubated for 4 h followed by the addition of 50 µL of DMSO per well, which were mixed thoroughly by a micropipette and left for 45 s. Presence of viable cells was visualized by the development of a purple color due to formation of formazan crystals. The assays were carried out in triplicates and repeated twice to ensure the reproducibility of results. The OD values were read at 570 nm using DMSO as a blank. A standard graph was plotted between the concentration of inhibitors and the relative cell viability, which was calculated as follows:

$${\rm{Cell}}\,{\rm{viability}}( \% )=\frac{{\rm{Test}}\,{\rm{OD}}}{{\rm{Control}}\,{\rm{OD}}}\times {\rm{100}}$$

### Entry and intracellular replication of *M. marinum* in cultured RAW264.7 and THP-1 cells

RAW264.7 and THP-1 macrophages were infected with WT *M. marinum*, ΔMelF and WT + inhibitors as described previously^12,41^ with some modifications. Briefly, murine macrophages were seeded in 24-well plates at a concentration of 5 × 10^5^ cells/well and the cells were activated overnight with 100 U/mL of murine IFN-γ (Sigma-Aldrich) prior to infection. A single-cell suspension of dispersed *M. marinum* growth (OD_600_ = 0.5–0.6) was used to infect the macrophages. For entry assays, an MOI of ~ 0.2 was employed and 1X MIC of inhibitors was added per well followed by infection with *M. marinum* for 30 min at 33 °C in 5% CO_2_ with humidified atmosphere. Following incubation, extracellular bacteria were removed by washing with warm medium (without FBS) and then 1 ml of 0.1% Triton X-100 was added/well. After rigorous pipetting, serial dilutions of lysates were plated for CFU enumeration. The assays were carried out in triplicates and repeated twice to ensure the reproducibility of results.

For intracellular replication/persistence experiments, 10^6^ human THP-1 monocytic cells were pre-treated with 10 ng/mL PMA overnight to result in adherent macrophages in 24-well plates and activated overnight with 100 U/mL of human IFN-γ (Sigma-Aldrich). Both activated RAW264.7 and THP-1 macrophages were infected with *M. marinum* at an MOI of ~1.0 and ~ 0.1, for intracellular replication and persistence experiments, respectively in 24-well plates for 30 min as described above, followed by washing with warm medium (without FBS) and addition of fresh medium + 1% FBS in presence of 1X and 5X MIC of inhibitors. In a similar manner, RAW264.7 and THP-1 macrophages were also infected with WT and ΔMelF, which served as positive and negative controls, respectively. Since ΔMelF displayed a significantly lesser cell association/entry within the RAW264.7 cells as compared to WT (Table 1), we infected the macrophages with ΔMelF at an MOI of ~1.8 and ~ 0.18 for intracellular replication and persistence experiments, respectively to get approximately the same numbers of bacteria on day 0 p.i. The synergistic effect of # 6513745 (at 1X MIC) in combination with INH at 1X MIC was also evaluated against the intracellular replication of *M. marinum* for 7 days within RAW264.7 macrophages. We included INH alone (6.25 µg/mL) and # 6513745 (6.25 µg/mL) alone in such experiments at their respective MIC. The 24-well plates were incubated at 33 °C in 5% CO_2_ with humidified atmosphere for intracellular replication and persistence experiments for 7 and 14 days, respectively. On every alternate day, the media was removed and replenished with fresh warm media containing the respective inhibitors (at 1X and 5X MIC). On different days p.i., the infected monolayers were washed with warm medium (without FBS) and lysed with 1 mL of 0.1% Triton X-100 for 5 min to release the intracellular mycobacteria. Lysis of macrophage monolayer was ascertained by viewing the 24-well plate under a phase-contrast microscope and the serial dilutions of lysates were plated for CFU enumeration. The assays were carried out in triplicates and repeated twice to ensure the reproducibility of results.

### Transmission electron microscopic (TEM) analysis

Murine IFN-γ activated RAW264.7 cells were infected with WT *M. marinum* in T-75 flasks at an MOI of ~1.0 in the absence and presence of # 5175552 (1X MIC). Similarly, RAW264.7 cells were infected with ΔMelF at an MOI of ~1.8 to get the same number of bacteria on day 0 p.i. Infected RAW264.7 cells were harvested on day 2, 4 and 7 p.i., washed thrice with PBS and fixed in freshly prepared 4% paraformaldehyde. The monolayers were incubated at RT for 30 min, washed thrice with PBS and post-fixed with 1% osmium tetraoxide in 0.1 M phosphate buffer at 4 °C for 1 h^19,42^. Samples were dehydrated with graded acetone, cleared with toluene and infiltrated with toluene and araldite mixture at 25 °C and then kept overnight at 50 °C in pure araldite. Later, the samples were embedded in 1.5 mL eppendorf tubes with pure araldite mixture at 60 °C for ultra-thin section cutting with the help of ultra-microtome (Ultra-microtome Leica EM UC6). Sections were placed on copper grid (3.05 mm diameter and 200 mesh), stained with uranyl acetate and observed at 120 kV under TEM (Model # JEOL2100F with accelerating voltage 80 kV–200 kV and Magnification 50 to 1,500,000X).

### Statistical analyses

Statistical analyses were carried out for cytotoxicity, entry, intracellular replication and persistence experiments. Two independent experiments were performed in triplicates assuming that the observations resulted from normal population and homogeneous variance. The average of these independent experiments along with the standard deviation (SD) was calculated for each assay. Student’s *t*-test was performed to check if there was any significant difference between the mean values of two groups. To show the significance and reproducibility of experiments, R software was employed for all analyses and *p-*value calculations^43^. A *p-*value of <0.05 indicated a significant difference between two independent groups of observations.

### Ethics approval and consent to participate

The Departmental committee of THSTI, Faridabad has given ethical approval to work on *M. marinum* M strain in biosafety-hood.

## Supplementary information

Supplementary Dataset 1

## Data Availability

All the data is available in the manuscript.
