# Supplementary material for: Evaluation of in silico designed inhibitors targeting MelF (Rv1936) against Mycobacterium marinum within macrophages
Source: Sci Rep. 2019 Jul 12;9:10084. doi: 10.1038/s41598-019-46295-5 (PMC6626058; doi:10.1038/s41598-019-46295-5)
Supplement: Supplementary file 1 — Supplementary Dataset 1 [file 41598_2019_46295_MOESM1_ESM.pdf]

Title:

Evaluation of *in silico* designed inhibitors targeting MelF (Rv1936) against *Mycobacterium marinum* within macrophages

Authors:

Renu Dharra[1], V.S. Radhakrishnan[2], Tulika Prasad[2], Zoozeal Thakur[1], Jeffrey D. Cirillo[3], Amit K. Pandey[4], Mahesh Kulharia[5] and Promod K. Mehta[1*]

Suppl. Table 1: Raw data for intracellular replication experiment

RAW264.7: CFU/mL versus time (days) at 1X MIC of inhibitor

	CFU/mL (0 hr)		CFU/mL (Day 2)		CFU/mL (Day 4)		CFU/mL (Day 7)	
Group	Expt 1 (mean)	Expt 2 (mean)	Expt 1 (mean)	Expt 2 (mean)	Expt 1 (mean)	Expt 2 (mean)	Expt 1 (mean)	Expt 2 (mean)
WT	145000	150000	612000	636000	3600000	3825000	2800000	2650000
Δ MelF	137600	130000	201000	205000	1400000	1470000	356000	380000
Rifampicin	154000	150000	306000	365000	1180000	1100000	556000	630000
WT + 5175552	103000	86000	506000	520000	1080000	1010000	17200	17000
WT + 5255829	156000	150000	1260000	1200000	1020000	1000000	188000	130000
WT + 6513745	152000	230000	169000	110000	40500	46500	34400	36000
WT + 9125618	122000	120000	1310000	1200000	1680000	1600000	1290000	1200000

RAW264.7: CFU/mL versus time (days) at 5X MIC of inhibitor

	CFU/mL (0 hr)		CFU/mL (Day 2)		CFU/mL (Day 4)		CFU/mL (Day 7)	
Group	Expt 1 (mean)	Expt 2 (mean)	Expt 1 (mean)	Expt 2 (mean)	Expt 1 (mean)	Expt 2 (mean)	Expt 1 (mean)	Expt 2 (mean)
WT	145000	150000	612000	636000	3600000	3825000	2800000	2650000
Δ MelF	137600	130000	201000	205000	1400000	1470000	356000	380000
Rifampicin	115000	160000	223000	300000	148000	280000	19000	20000
WT + 5175552	161000	180000	15000	23000	3000	3000	5500	6400
WT + 5255829	137000	260000	29100	34000	4700	4000	20200	16000
WT + 6513745	91000	90000	18000	16100	1000	1190	3500	4000
WT + 9125618	134600	132000	61200	47000	8300	15000	85000	70000

THP-1: CFU/mL versus time (days) at 1X MIC of inhibitor

	CFU/mL (0 hr)		CFU/mL (Day 2)		CFU/mL (Day 4)		CFU/mL (Day 7)	
Group	Expt 1 (mean)	Expt 2 (mean)	Expt 1 (mean)	Expt 2 (mean)	Expt 1 (mean)	Expt 2 (mean)	Expt 1 (mean)	Expt 2 (mean)
WT	200000	195000	270000	276000	900000	1044000	1380000	1370000
Δ MelF	200000	207000	130000	138000	260000	280000	170400	160000
Rifampicin	201500	201000	90000	90000	160000	177000	130000	134000
WT + 5175552	210000	225000	30000	31300	60000	54800	26000	22000
WT + 5255829	201500	199000	50000	49000	165000	173000	200000	205000
WT + 6513745	200000	209000	80000	68000	17000	10900	12000	14300
WT + 9125618	220000	210000	95000	98000	700000	736000	280000	288000

THP-1: CFU/mL versus time (days) at 5X MIC of inhibitor

	CFU/mL (0 hr)		CFU/mL (2 Days)		CFU/mL (4 days)		CFU/mL (7 Days)	
Group	Expt 1 (mean)	Expt 2 (mean)	Expt 1 (mean)	Expt 2 (mean)	Expt 1 (mean)	Expt 2 (mean)	Expt 1 (mean)	Expt 2 (mean)
WT	200000	195000	270000	276000	900000	1044000	1380000	1370000
Δ MelF	200000	207000	130000	138000	260000	280000	170400	160000
Rifampicin	201500	201000	84000	110000	58800	61000	171000	130000
WT + 5175552	210000	225000	16600	19000	39400	59000	51000	40000
WT + 5255829	201500	199000	12500	16000	12100	9000	27000	30000
WT + 6513745	200000	209000	3000	3000	3600	7000	23200	20000
WT + 9125618	220000	210000	31000	50000	14000	10000	153000	170000

Suppl. Table 2: Raw data for 14 days persistence experiment

RAW264.7: CFU/mL versus time (days) at 1X MIC of inhibitor

	CFU/mL (0 hr)		CFU/mL (Day 4)		CFU/mL (Day 7)		CFU/mL (Day 10)		CFU/mL (Day 14)	
Group	Expt 1 (mean)	Expt 2 (mean)	Expt 1 (mean)	Expt 2 (mean)	Expt 1 (mean)	Expt 2 (mean)	Expt 1 (mean)	Expt 2 (mean)	Expt 1 (mean)	Expt 2 (mean)
WT	30000	32000	84000	90000	480400	495900	350000	289000	265000	249000
Δ MelF	30800	27000	46000	31000	79800	76100	84100	74900	22100	24900
Rifampicin	25500	24000	59000	66000	84000	11800	42800	50000	11200	10100
WT + 5175552	22000	23200	58000	60000	8500	7000	2110	3600	4100	3000
WT + 5255829	21200	22300	32000	28000	59000	61000	15800	18000	130000	189000
WT + 6513745	20000	27100	8400	16000	7000	6500	35000	36100	41500	43200
WT + 9125618	24500	23200	23600	20000	97800	125000	117000	99000	15200	9000

RAW264.7: CFU/mL versus time (days) at 5X MIC of inhibitor

	CFU/mL (0 hr)		CFU/mL (Day 4)		CFU/mL (Day 7)		CFU/mL (Day 10)		CFU/mL (Day 14)	
Group	Expt 1 (mean)	Expt 2 (mean)	Expt 1 (mean)	Expt 2 (mean)	Expt 1 (mean)	Expt 2 (mean)	Expt 1 (mean)	Expt 2 (mean)	Expt 1 (mean)	Expt 2 (mean)
WT	30000	32000	84000	90000	480400	495900	350000	289000	265000	249000
Δ MelF	30800	27000	46000	31000	79800	76100	84100	74900	22100	24900
Rifampicin	25500	24000	610	900	13800	14000	840	700	1720	2500
WT + 5175552	22000	23200	800	1060	3900	8000	170	200	330	390
WT + 5255829	21200	22300	840	1400	710	500	1590	1800	3440	2700
WT + 6513745	20000	27100	340	300	1900	2000	1610	2000	2700	2810
WT + 9125618	24500	23200	1200	2000	4230	5700	4680	6100	1600	2000

THP-1: CFU/mL versus time (days) at 1X MIC of inhibitor

	CFU/mL (0 hr)		CFU/mL (Day 4)		CFU/mL (Day 7)		CFU/mL (Day 10)		CFU/mL (Day 14)	
Group	Expt 1 (mean)	Expt 2 (mean)	Expt 1 (mean)	Expt 2 (mean)	Expt 1 (mean)	Expt 2 (mean)	Expt 1 (mean)	Expt 2 (mean)	Expt 1 (mean)	Expt 2 (mean)
WT	25500	36000	64000	55000	402000	412000	350000	465000	356000	315000
Δ MelF	21600	27000	46000	30000	84000	81000	65000	64100	33000	41000
Rifampicin	25500	24000	9000	8900	84000	90000	40800	41100	52000	62000
WT + 5175552	22000	23200	28000	58000	8500	7000	2110	3600	4100	3000
WT + 5255829	21200	22300	17600	32000	59000	62000	15800	18000	230000	200000
WT + 6513745	20000	27100	8400	16000	5950	3910	31000	35500	35000	33500
WT + 9125618	24500	23200	23600	20000	97800	125000	111000	117000	15200	9000

THP-1: CFU/mL versus time (days) at 5X MIC of inhibitor

	CFU/mL (0 hr)		CFU/mL (Day 4)		CFU/mL (Day 7)		CFU/mL (Day 10)		CFU/mL (Day 14)	
Group	Expt 1 (mean)	Expt 2 (mean)	Expt 1 (mean)	Expt 2 (mean)	Expt 1 (mean)	Expt 2 (mean)	Expt 1 (mean)	Expt 2 (mean)	Expt 1 (mean)	Expt 2 (mean)
WT	25500	36000	64000	55000	402000	412000	350000	465000	356000	315000
Δ MelF	21600	27000	46000	30000	84000	81000	65000	64100	33000	41000
Rifampicin	25500	24000	620	700	17800	19000	840	700	3110	3020
WT + 5175552	22000	23200	1440	2200	3900	8000	170	100	133	128
WT + 5255829	21200	22300	780	800	710	500	1590	1800	3440	2700
WT + 6513745	20000	27100	910	1000	1570	1900	1610	2000	3200	4300
WT + 9125618	24500	23200	660	1200	4230	5700	4680	8100	1600	2000
